# Motion Extrapolation in the Central Fovea

**DOI:** 10.1371/journal.pone.0033651

**Published:** 2012-03-15

**Authors:** Zhuanghua Shi, Romi Nijhawan

**Affiliations:** 1 Department Psychologie, Ludwig-Maximilians-Universität München, Munich, Germany; 2 School of Psychology, University of Sussex, Brighton, United Kingdom; University of California, Berkeley, United States of America

## Abstract

Neural transmission latency would introduce a spatial lag when an object moves across the visual field, if the latency was not compensated. A visual predictive mechanism has been proposed, which overcomes such spatial lag by extrapolating the position of the moving object forward. However, a forward position shift is often absent if the object abruptly stops moving (motion-termination). A recent “correction-for-extrapolation” hypothesis suggests that the absence of forward shifts is caused by sensory signals representing ‘failed’ predictions. Thus far, this hypothesis has been tested only for extra-foveal retinal locations. We tested this hypothesis using two foveal scotomas: scotoma to dim light and scotoma to blue light. We found that the perceived position of a dim dot is extrapolated into the fovea during motion-termination. Next, we compared the perceived position shifts of a blue versus a green moving dot. As predicted the extrapolation at motion-termination was only found with the blue moving dot. The results provide new evidence for the correction-for-extrapolation hypothesis for the region with highest spatial acuity, the fovea.

## Introduction

A moving object appears to be ahead of a spatially aligned flashed object. This phenomenon, termed the flash-lag effect, has been addressed in over a hundred articles in the last decade and a half and several hypotheses have been proposed to explain it (see reviews [1]–[5]). The initial hypothesis proposed by Nijhawan [6] suggested that the position of the moving object is extrapolated forward to compensate for neural delays in the visual pathway so the object's perceived position is closer to the object's true instantaneous location. The differential latency [7]–[9] and the attention shift [10] hypotheses assumed the moving object has shorter afferent delay than the flash; the temporal integration hypothesis suggested the perceived position is an average of sampled positions of a moving object over an extended period of time [11], [12] and the Postdiction account proposed that the flash resets motion integration and the position of a moving object is determined about 80 ms after the flash onset [13], [14].

This area is hotly debated. The initial experimental results that contributed to the debate were based on the so-called flash-terminated and flash-initiated conditions of the flash-lag effect. The counter-intuitive results were that the flash-terminated condition, in which motion is only visible before the flash (i.e. there is no motion input following the flash), produced no flash-lag effect, while the flash-initiated condition, in which motion is only visible following the flash (i.e. there is no motion input before the flash), produced a full-fledged flash-lag effect [15]. In the past decade, several articles have underscored the importance of these results [2], [3], [5], [7], [11], [14], [16].

A promising explanation of the flash-lag effect is one that considers not just the fact that natural motion, over short durations and distances, is predictable but also that given natural constraints, predictions can often fail; to appreciate these facts one need, in the first instance, only consider inertia and occluding property of opaque objects. As an example of failed prediction consider a prey that runs at first in a straight line, and then to dodge the predator it abruptly stops or changes direction at a sharp angle. It is likely that predictable events and unpredictable perturbations of such events are coded by different neural mechanisms. Indeed, predictable events may be coded by the ‘silence’ of neurons [17], [18], while failed predictions are “communicated loudly” by synchronous neural bursts in the early visual pathway [19], [20].

Our view is that both types of mechanisms serve important visual localization function, and together they reduce the overall position errors [2], [3]. A neural model for localization that accommodates these requirements is the “biased competition” model [21]. According to this model, in common sensorimotor interactions, two (sometimes multiple) competing neural representations could, initially, exist. The animal's nervous system must then favor one representation at the expense of the other(s) before it can act. Neural activity of the favored representation is augmented while that of the non-favored one is suppressed. In the flash-terminated condition of the flash-lag effect the predictive representation is suppressed and overwhelmed by the signals due to motion-termination, and consequently the moving object is not seen in the forward shifted position [2], [3], [22]–[24]. It is important to note, however, that the suppression by signals due to failed predictions is likely to be achieved shortly after the ‘stop’ signal due to neural latency. So, before the suppression there would be a small forward shift due to the previously set up predictive representation. However, due to weakened extrapolation and the masking effect resulting from two competing representations, such forward shift is not observed (see [2], Fig. 4 for a graphic description of this).

In animals that have foveas, these specialized anatomical loci play a vital role during sensorimotor behavior requiring precise localization. However, a study of the biased competition model for the localization function of the fovea has not been carried out thus far. Despite its importance and remarkable capabilities, an equally remarkable fact is that the human fovea has two scotomas. One scotoma is observed commonly when one looks directly at a dim object, such as a star. Although it is clearly seen in peripheral vision, the star vanishes on direct inspection. This is because the density of the highly sensitive rods drops sharply near the fovea and there are no rods in the central one degree of the fovea area [25]. A lesser-known scotoma is the disappearance of a blue object when viewed foveally. This is because of yellow macular pigment and low density of short wavelength cones in central fovea [26]. The pigmented area absorbs blue light, which leads to the well-known entoptic phenomena of Haidinger's brushes and Maxwell's spot ([27], also see Video S1).

These scotomas provide almost ideal conditions to test the correction-for-extrapolation mechanisms [2], [3] in the motion-terminated condition. First, the fovea is associated with highest spatial acuity and correction-for-extrapolation is, first and foremost, a spatial hypothesis. Secondly, test of blue scotoma can be elegantly accomplished by comparing very similar stimuli that should, according to the hypothesis, behave very differently. Previous research has shown perceptual filling-in and inhibition of border at a scotoma [28], [29]. The transient signal induced by the motion-termination inside the scotoma or at its boundary would be relatively weaker than that in the normal visual field. Thus the predictive representation at motion-termination in the scotoma may not be fully suppressed by the stop signal and consequently become visible.

When a dim object moves into the rod-free area, its position signal is weak or even absent. According to the correction-for-extrapolation hypothesis [2], [3], a dim object moving into the fovea should be extrapolated into the light *insensitive* foveal areas, and be seen in those retinal positions even though the physical stimulus energy there is insufficient to yield a percept. A similar effect should be found with a blue object, but not with a green object to which the fovea should be relatively more sensitive (see Video S1). Thus in this study we employ a dim moving object (Experiment 1) and blue/green objects (Experiment 2) to study the correction-for-extrapolation hypothesis in the central fovea.

## Methods

### Participants

Six separate participants volunteered for Experiment 1 (3 females) and Experiment 2 (4 females). All participants had normal vision. Written informed consent was obtained before the experiment. The experiments have been approved and permitted by the Ethics Commission, Institute for Psychology and Education, Ludwig-Maximilians-Universität München, Germany.

### Stimuli and procedure

Experiments took place in a dark room. The participant sat in front of the monitor with viewing distance of 52 cm. The dominant eye was aligned with the center of the screen and monitored by a head supported eye Tracker (EyeLink 1000). After the calibration of the eye tracking system, the participant rested for about 20 minutes for dark adaptation.

**Figure 1:**
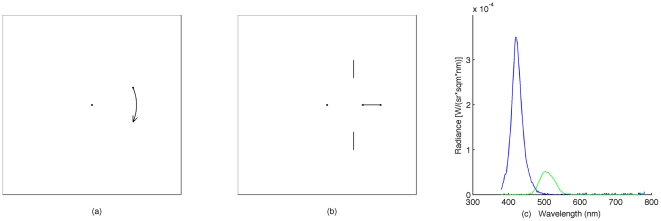
Schematic illustration of the stimuli used in the experiments. (a) Motion detection task. A dot appears on the left or the right side of the fixation point (FP) at a given eccentricity and revolves around the fixation for 100 ms. (b) Movement localization task. A dot moves from the left or the right towards the fixation point (motion-terminated condition) or moves away from fixation to the left or the right side (motion-initiated condition). (c). Spectral charts for the blue filter (blue curve) and the green filter (green curve). The scotopic luminance for the blue and the green was approximately 3.93 cd/m^2^ (used for the fixation and reference bars) calculated with the scotopic luminosity function (CIE 1951).

In Experiment 1, a neutral density plastic filter (LEE filter, reducing light 4 stops) was attached on the surface of the screen to reduce the luminance. The experiment consists of two sessions: the motion detection and the moving object localization (Figure 1).

In the motion detection session, a trial started with a small dim fixation point (diameter: 0.17°; luminance: 0.28 cd/m^2^) and a warning tone (100 ms, 1000 Hz, 63 dB). The positions of the eye were monitored online. After random interval (300–500 ms), provided the position of eye had not deviated from the fixation point by more than 0.5 deg, a small dim dot (diameter: 0.17°; luminance: 0.028 cd/m^2^) appeared on the left or the right at a given eccentricity (7 levels, from 0.5° to 2.3° with steps of 0.3°) and revolved around the fixation at 5.0 radians/sec for 100 msec. The participant then had to indicate if he/she saw the rotating dot. Each eccentricity condition was repeated 24 times and counter-balanced on the left and the right sides and the direction of the motion. In addition, 14 catch trials (i.e. with no moving dot) were randomly mixed with the other trials.

In the moving object localization session, a trial started with the presentation of the fixation point (diameter: 0.17°; luminance: 0.28 cd/m^2^) and the two vertical collinear reference lines (subtending: 0.08°×0.41°; luminance: 0.28 cd/m^2^) for 300–500 ms. The vertical positions of the reference lines were 1.2° above and below the fixation point. The horizontal position of the reference lines was varied from trial to trial (see further details below). When the eye was fixated on the fixation point (online, measured by eye tracker, and the deviance was less than 0.5°), another dim dot (diameter: 0.17°; luminance: 0.028 cd/m^2^) appeared. On half the trials, the dot started to move (at 5°/sec) from a position 8° to the left or the right of fixation towards the fixation point and vanished at the center (the *motion-terminated condition*, see Figure 1b). The participant had to indicate if the moving dot vanished to the right or to the left of the reference lines, which were positioned randomly between 0° and 1.8° with a step size of 0.3° away from the fixation point (on the same side as the movement). On the other half of the trials, the dim dot started to move from the center to the left or the right and vanished at the 8° position (the *motion-initiated condition*). The task was to indicate if the moving dot's first perceived position was to the left or the right of the reference lines. In this case the horizontal position of the reference lines was randomly chosen from 0.5° to 2.3° with steps of 0.3°. The range of the reference positions was chosen based on a pilot experiment. The motion-terminated condition and the motion-initiated condition were run in separated blocks, each with 28 trials. The order of the blocks was randomized. Each condition contained 7 levels of reference positions, which were randomly repeated 20 times and the left/right visual field presentations were counterbalanced.

In Experiment 2, the stimuli and procedure were the same as in Experiment 1, excepting the following differences: A blue (Tokyo blue LEE filter, dominant wave length: 422 nm) or a green (Primary green LEE filter, dominant wave length: 501 nm) plastic filter was attached on the surface of the monitor in separated sessions. We used the cyan color for all stimuli on the screen to reduce the red spectrum. The spectral characteristics of the two filters for a cyan color on the screen are illustrated in Figure 1c. The intensities of the stimuli were adjusted for the two filters separately such that both scotopic luminances were approximately equal. The fixation point and reference lines were set to 3.92 cd/m^2^ (radiance shown in Figure 1c) and the moving/revolving dots were set to 0.95 cd/m^2^ (Measured by JETI spectrometer and calculated with the scotopic luminosity function, CIE, 1951). The motion detection task was run only in the blue filter condition since the detection of the green revolving dot was far above the threshold with the given luminance.

### Data analysis

Participants' responses were first converted to proportions of visibility for the detection task, the motion-initiated and motion-terminated localization tasks. Psychometric curves were then fitted using a logistic function to each condition and points of subjective equality (PSEs) were estimated from the 50% point of corresponding psychometric function.

## Results

### Experiment 1: using dim moving dot

**Figure 2:**
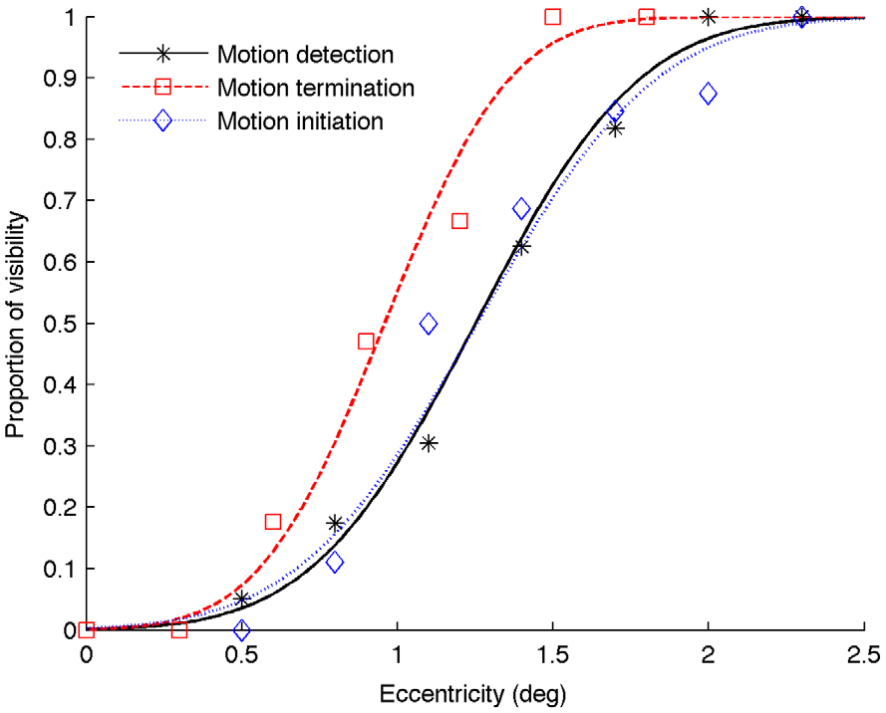
Psychometric curves for a typical participant from Experiment 1. The black solid curve (with stars) represents the proportion the revolving dot is detectable at given eccentricity. The red dashed curve (with squares) represents the proportion the moving dot is seen at given eccentricity in the motion-terminated condition. The blue dotted curve (with diamonds) denotes the proportion the moving dot is seen at given eccentricity in the motion-initiated condition.

**Figure 3:**
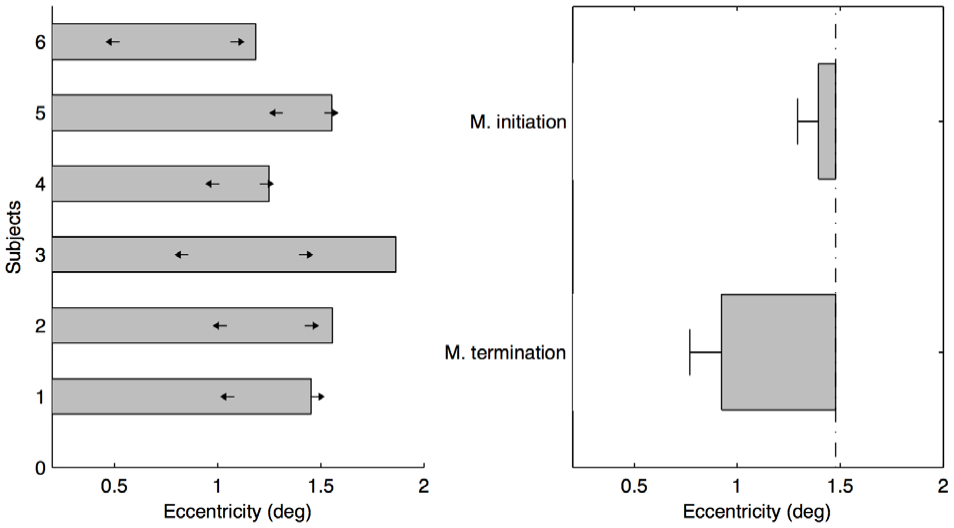
Results of Experiment 1. (a) Individual thresholds of participants for three conditions. The left arrows denote the perceived vanishing positions in the motion-terminated condition; the right arrows denote the perceived initial positions in the motion-initiated condition; the gray bars denote the thresholds (50%) of motion visibility at 0.028 cd/m^2^. (b) Mean forward shifts in the motion-initiated and motion-terminated conditions (±SE, n = 6). The vertical dot-dashed line denotes the mean radius of the relatively insensitive fovea centralis.

All participants exhibited low rates of false alarms (mean: 1.2%) in the catch trials. Psychometric curves for one typical observer are shown in Figure 2. The thresholds of the perceived initiation, termination and the boundary of motion insensitive fovea area for all participants are shown in Figure 3a. The mean threshold (±standard error, SE) measured in the motion detection task (duration of 100 ms) was 1.48±0.11° (indicated by the vertical dot-dashed line in Figure 3b), which represents the boundary of the motion insensitive fovea center for the given luminance (0.028 cd/m^2^). Inside the motion insensitive fovea area, the estimated mean detectability of the revolving dot at the eccentricity 0.5°, which is regarded as in the rod-free area according to the anatomical size [25], was 2.5%, as low as the mean false alarm rate (t(5) = 0.76, p = 0.48, η_p_^2^ = 0.1). This suggested that in the rod-free area there was no response to the low luminance motion.

Figure 3a shows that all participants perceived the moving dot as vanishing inside the motion insensitive fovea center in the motion-terminated condition and appearing near the boundary of the motion insensitive area in the motion-initiated condition. The mean perceived termination and initiation positions (±SE) were 0.92±0.12° and 1.39±0.07°, respectively. Compared with the boundary of the motion insensitive fovea center, the average forward shift into the boundary was 0.55±0.13° (corresponding to 110.9±26.3 ms) in the motion-terminated condition [t-test: *t*(5) = 4.61, *p*<0.01, η_p_^2^ = 0.81.]. Even compared with the anatomical boundary of the rod-free area (about the eccentricity 0.5°, [25]), the mean proportion of vanishing position inside the eccentricity 0.5° was 21.1±7.2%, significantly higher than the motion detection level (2.5%) [t(5) = 2.67, p<0.05, η_p_^2^ = 0.59].

In the classical Fröhlich effect [30]–[32], the forward shifts in the motion initiation are often measured relative to a static reference. If we considered the physical initial position (i.e. the fixation point), we had huge classical Fröhlich effect, 1.39±0.07°, *t*(5) = 19.8, p<0.001, which was mainly because the initial movement region was motion insensitive foveal center. However, if we used the motion detection threshold (50%) as a relative boundary reference, there was no significant shifts in the motion-initiated condition [t-test: *t*(5) = 1.2, *p* = 0.28, η_p_^2^ = 0.22.]. The rotatory motion threshold estimated by the 50% of the psychometric curve could potentially reduce our measurement of the forward shifts. Another possible factor could be the motion direction used in the motion-initiated condition (i.e. the foveofugal motion). It has been shown that the foveofugal motion produced less strong movement mislocalization [33].

### Experiment 2: using blue and green moving dots

**Figure 4:**
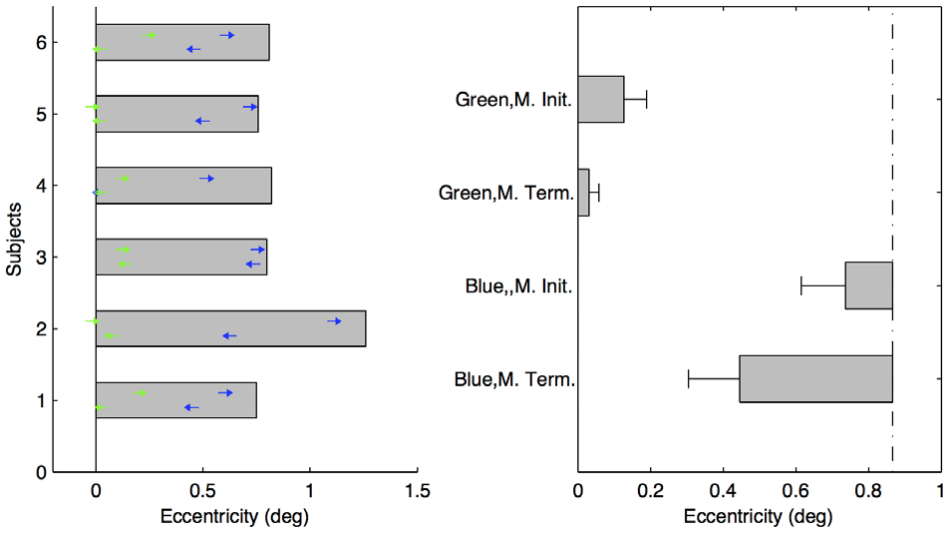
Results of Experiment 2. (a) Individual thresholds for four conditions. The left arrows denote the perceived vanishing positions in the motion-terminated condition; the right arrows the perceived initial positions in the motion-initiated condition; The blue arrows represent the thresholds with the blue filter and the green arrows with the green filter. The gray bars denote the motion detection threshold for the blue moving object. (b) Mean forward shifts for four conditions (±SE, n = 6). The vertical dot-dashed line represents the mean threshold of motion detection.

The method of estimating the initiation positions, the termination positions and the boundary of the motion insensitive area was the same as in Experiment 1. There were no false alarms in the catch trials. Figure 4a shows the thresholds for all participants with the green and the blue filters. The motion insensitive boundary estimated with the blue filter was 0.87±0.09° (indicated by the vertical dot-dashed line in Figure 4b), which agreed with previous estimates of Maxwell's spot [26], [27]. The blue moving dot was perceived to vanish at position 0.45±0.11° in the motion-terminated condition, and to first appear at position 0.74±0.09° away from the center in the motion-initiated condition.

Using the motion insensitive boundary, we calculated the positional shifts of the blue moving dot (Figure 4b). Consistent with the result for the dim moving dot (Experiment 1), the blue moving dot overshot significantly into the motion insensitive boundary by 0.42±0.12° (corresponding to 86±24 ms) in the motion-terminated condition, *t*(5) = 3.83, *p*<0.05, η_p_^2^ = 0.75. The classical Fröhlich effect (compared with the physical initial position) was significant, t(5) = 10.87, p<0.001. However, the magnitude of shift in the motion-initiated condition was not significant when compared with the motion insensitive boundary, *t*(5) = 2.48, *p* = 0.07, η_p_^2^ = 0.51.

In contrast to the results for the blue moving object, we obtained results consistent with the classical flash-lag effects in the flash-terminated condition for the green moving dot (using the green filter) with the same scotopic luminance. There was no evidence of forward shift in the motion-terminated condition, t(5) = 1.63, p = 0.16, η_p_^2^ = 0.35, while a significant forward shift (i.e. Fröhlich effect) was found in the motion-initiated condition, [mean shift: 0.13±0.05°, t(5) = 2.85, p<0.05, η_p_^2^ = 0.62].

## Discussion

When a moving object disappears unpredictably, simultaneously with an aligned flash, there is no flash-lag effect [2], [14], [15]. There has long been a debate over what causes the absence of the flash-lag effect in the motion-terminated condition (see review [3]). Most proposed accounts (e.g. differential latency [7]–[9], moving average [11], [12], and postdiction [13], [14]) rely on the relationships between the flash and the moving object and argue that the motion after the flash onset (or motion stop) plays an important role in the flash-lag effect. These accounts argue that the absence of the motion after the flash onset (or motion stop) in the motion-terminated condition leads to the absence of the flash-lag effect [7], [8], [13], [14]. In contrast, the correction-for-extrapolation hypothesis suggests that strong transient signals triggered by the stopping of the moving object *itself* provide a correction signal for the forward shift [2], [3]. This is independent of the presence of the flash. It was recently reported that abrupt direction change is signaled strongly by the early visual system [19], [20]. Our proposed correction process acts only after a brief period of the external stop signal, due to the neural transmission latency, so one might expect a short-lived overshoot during the latency period. However, retroactive impact of later events on earlier events is well known in cases such as backward masking (where the second stimulus masks the first). In this case, although the first signal is present on its own for a brief duration, it is nonetheless rendered completely invisible.

In our account, the signal corresponding to the extrapolated position is quickly followed by a signal from the retina representing direction change and the position tag of this signal is veridical. The extrapolated signal is masked by the direction change signal rendering the former unavailable for reporting (see [2], Fig. 4 for a graphic outline of this process). According to our account, by weakening the transient signals [22] or eliminating them (i.e. reducing the suppression), as when an object moves into the physiological blind spot [24], forward shifts during motion-termination become manifest again.

Here we tested the correction-for-extrapolation hypothesis in the central fovea without the use of a flash. The main comparison was between the boundary of the motion insensitive area and the termination position of a horizontally moving dot traveling toward the fovea. Our finding was that a dim moving dot shifted into the motion insensitive foveal area by about 0.55° (corresponding to 111 ms) and a blue moving dot shifted into the Maxwell's spot by about 0.42° (corresponding to 86 ms). Moreover, the probability of a dim moving dot extrapolated into the rod-free area at 0.5° was significant higher than the chance level. In contrast, the apparent termination position of a green object, an otherwise comparable stimulus to the blue object, was close to veridical. The different behavior of the blue and green moving dots provides new support for the correction-for-extrapolation hypothesis.

In the motion-initiated condition, we found the classical Fröhlich effect [30]–[32] for all conditions (compared with the static fixation). Interestingly, the perceived initial position of the foveofugal movement was close to the boundary of the motion insensitive area for the dim and blue moving dots. If we consider the boundary as the reference position, there was no Fröhlich effect. This could be partially because the motion insensitive boundary is not an on/off step (indicated by the psychometric curve), which could have led to some degree of underestimation of the forward shift. In addition, less strong movement mislocalization when motion is away from the fovea could have contributed to this [33].

As we did not employ a flash as a reference, our results cannot be explained by the differential latency account [7]–[9] since it would need the static flash as a reference. Postdiction account [13], [14] and the moving average account [11], [12] would rely on the motion information after the flash (or a stop signal) and so would also predict no forward shifts for the motion-terminated condition, contrary to our results obtained with the dim and blue moving objects. Our findings underscore the importance of the biased competition model in evaluating the results of the flash-terminated condition. One of the defining features of this model, which has been previously used to address phenomena such as visual attention, is that a new feature appearing in the environment is given greater relative weight during the competitive neural interactions [21].

We have argued elsewhere that instead to evolving the fastest possible reactions to stimulation, the primate visual system has evolved mechanisms that require delays [34]. Two good examples are: 1) spatiotemporal integration (e.g. at the level of retinal ganglion cells), which allows for a high degree of light sensitivity. This mechanism is of necessity time-consuming, and 2) computations carried out by motion detectors (e.g. Reichardt detector) that explicitly depend on time delays. The computational benefits introduced by delays, however, also confer a potential drawback, leading to spatial lags during the animal's interaction with moving objects [35], [36]. Compensation for the delays is the biological process that removes this disadvantage. The correction-for-extrapolation hypothesis proposes a further step that corrects for the compensation, when strongly signaled by the receptors, to minimize the overall spatial errors.

Two general considerations further support this hypothesis. The first is related to predictions/failed-predictions, and the other to response competition. It is often seen in predator-prey interactions that if the prey cannot outrun the predator then it attempts to produce unpredictable movements such as jumping, stopping or changing directions. From a predator's point of view, appropriate reaction to the prey on the basis of smoothly changing input (prediction), is as important as reacting appropriately to the prey's abrupt movements that violate those predictions. As an extreme example consider a prey animal with the ability to change shape and/or skin pattern for camouflage (e.g. cuttlefish) that has stopped moving, and has consequently become invisible to the pursuing predator. Locating such an animal in its last seen position, when it was still in motion, will obviously be beneficial to the predator. In this case the memory for the strongly signaled position where the animal stopped, and not the animal's extrapolated position would serve the predator better. On the other hand, using dazzle coloration or flicker-fusion camouflage to induce fake stopping signal would benefit the prey [37], [38].

It has been argued that what an animal will perceive and how it will react depends on the outcome of competing, mutually suppressive, neural interactions. In the case of compensation for delays during motion two neural representations exist. One compensates for the delays and exists throughout the smooth phase of motion, while the other is set up quickly following the failed-prediction signal triggered by abrupt direction change [19], [20]. The latter representation is stronger (see [21] for criteria) and consequently wins the competition during motion-termination. In the present experiments we weakened the representation of failed-prediction signal, and as a consequence the extrapolated representation wins the competition and reveals itself in perception.

## Supporting Information

Video S1 Requirements and what to see: The demo video requires an additional blue filter with peak wavelength around 450 nm (e.g. the LEE filter – Tokyo blue). Please wear the color filter glasses and fixate on the center fixation point. In the first part of the movie, a dot moves continuously leftward and rightward crossing the fixation point. Viewing through the blue filter, you may see that the moving dot approaches and vanishes near the fixation point, and reappears further away from the opposite side of the fixation. The invisible gap between the reappearing position and the fixation, which you may perceive, is larger than the gap between the vanishing position and the fixation point. Without the filter or with green filter glasses, you may see continuous movement, or the moving dot approaches to the fixation point and a small gap after the moving dot crossing the fixation point (known as Fröhlich effect). In the second part of the movie, a bright disk flashes at 1 Hz. Viewing through the blue filter, you may perceive a dark irregular ‘ink’ spot (about one to two degree of visual angle, known as Maxell's spot) surrounding your fixated area. The irregular dark spot is due to the fact that yellow macular pigment absorbs the blue light and relatively low density distribution of short wavelength cones in central fovea.
(MOV)
